# Draft Genome Sequence of *Moraxella bovoculi* Strain 237^T^ (ATCC BAA-1259^T^) Isolated from a Calf with Infectious Bovine Keratoconjunctivitis

**DOI:** 10.1128/genomeA.00612-14

**Published:** 2014-06-26

**Authors:** Michael J. Calcutt, Mark F. Foecking, Neal T. Martin, Tendai Mhlanga-Mutangadura, Thomas J. Reilly

**Affiliations:** a Department of Veterinary Pathobiology, University of Missouri, Columbia, Missouri, USA; b Veterinary Medical Diagnostic Laboratory, Department of Veterinary Pathobiology, University of Missouri, Columbia, Missouri, USA

## Abstract

*Moraxella bovoculi* is a recently identified species, recovered from the bovine eye, which is under investigation as an etiological agent of infectious bovine keratoconjunctivitis. A draft genome sequence of the *Moraxella bovoculi* type strain 237^T^ has been determined to identify features that may be important during host colonization.

## GENOME ANNOUNCEMENT

Infectious bovine keratoconjunctivitis (IBK) is a highly contagious disease of cattle, with annual losses exceeding $150 million (1). Historically, the disease was believed to be under the sole purview of *Moraxella bovis* (2); however, a new species, designated *Moraxella bovoculi*, was isolated from calves with IBK in 2002 (3). Although circumstantial evidence implicates a role in pathogenesis, the precise contribution of *M. bovoculi* to IBK development, whether as a primary pathogen, opportunist, or additional predisposing factor, has yet to be established (4).

A draft genome sequence of the type strain was determined to provide insight into the lifestyle of this ocular inhabitant and to identify molecular epidemiological targets. Genomic DNA from *M. bovoculi* 237^T^ (obtained as ATCC BAA-1259^T^) was sequenced using 454 technology at the Genome Institute, Washington University, St. Louis. Newbler assembly resulted in 43 chromosomally derived contigs comprising 2,043,923 bp and two novel cryptic plasmids (3.5 and 4.5 kb), with 66-fold overall coverage. The G+C content is 45.62%. The assembly of the large contigs was confirmed by optical mapping of AflII restriction fragments (Opgen, Gaithersburg, MD). The resulting assembly was auto-annotated using the PGAP pipeline at NCBI prior to PCR and Sanger sequence confirmation of 27 pseudogenes with frameshift mutations, deletions, or premature stop codons. The deduced gene set comprises 1,975 genes, including 1,900 coding sequences (CDS) and 43 tRNAs.

Analysis of genes with potential importance for host colonization revealed a single pilin-encoding gene, a situation dissimilar to that in *M. bovis* and *Moraxella lacunata*, wherein a site-specific inversion system orchestrates phase variation between two pilin genes (5, 6). The *M. bovoculi* pilin sequence is only 38% identical to the most similar pilin reported for *M. bovis*, suggesting that *M. bovis* pilin-based bacterins (7) would provide limited to minimal protection against IBK. A homolog of the bifunctional *M. catarrhalis* McaP adhesin and phospholipase B (8) is also present as one of four identified autotransporters. Iron acquisition systems include two members of the transferrin/lactoferrin-binding lipoprotein family and two putative iron-transporting ABC transporters. As anticipated, a single operon encoding an RTX toxin and associated export machinery is present (9).

A BRO β-lactamase gene that is 95% identical to that of *M. catarrhalis* is present, inserted between *gatA* and *gatB*. The incursion of *bro* genes into the *gatAB* intergenic region in *M. catarrhalis* has led to the rapid emergence of BRO-expressing clones (10) via transformation-mediated horizontal transfer (11). The presence of *bro* in *M. bovoculi* may also reflect lateral gene transfer but the source of the gene is not immediately apparent; PCR and sequence analysis demonstrate that this gene is not present in the *gatAB* locus of either *M. bovis* Epp63 or the *Moraxella ovis* type strain (unpublished data).

The annotated data set presented here is the first for this species and should augment future study of the role of this agent in IBK development in addition to providing resources for molecular typing and genetic manipulation.

### Nucleotide sequence accession number.

This whole-genome shotgun project has been deposited at DDBJ/EMBL/GenBank under the accession number AOMT00000000.
